# Bidirectional Interactions Between the Gut Microbiota and Incretin-Based Therapies

**DOI:** 10.3390/life15060843

**Published:** 2025-05-23

**Authors:** Vincenzo Trapanese, Annamaria Dagostino, Maria Resilde Natale, Federica Giofrè, Clara Vatalaro, Melania Melina, Francesca Cosentino, Silvia Sergi, Felice Imoletti, Rocco Spagnuolo, Franco Arturi

**Affiliations:** 1 Internal Medicine Unit, Department of Medical and Surgical Sciences, “Magna Graecia” University of Catanzaro, 88100 Catanzaro, Italy; annamaria.dagostino94@gmail.com (A.D.); resilde94@gmail.com (M.R.N.); federica.giofre@gmail.com (F.G.); clara.vatalaro@gmail.com (C.V.); m.melania87@gmail.com (M.M.); francescacosentino13@gmail.com (F.C.); silvia.sergi14@gmail.com (S.S.); arturi@unicz.it (F.A.); 2 Department of Health Sciences, “Magna Graecia” University of Catanzaro, 88100 Catanzaro, Italy; felice.imoletti@hotmail.it (F.I.); spagnuolo@unicz.it (R.S.); 3 Research Centre for the Prevention and Treatment of Metabolic Diseases (CR METDIS), “Magna Graecia” University of Catanzaro, 88100 Catanzaro, Italy

**Keywords:** gut microbiota, type 2 diabetes mellitus, obesity, incretin, DPP-4 inhibitor, GLP-1 receptor agonist, GLP-1/GIP receptor dual agonist

## Abstract

Obesity, insulin resistance, type 2 diabetes mellitus (T2DM) and metabolic syndrome have been largely correlated to a reduction in bacterial load and diversity, resulting in a condition known as intestinal dysbiosis. The recent emergence of novel antidiabetic medications has been demonstrated to exert a favourable influence on the composition of the intestinal microbiota. Incretin-based therapy exerts a multifaceted influence on the composition of the gut microbiota, leading to alterations in bacterial flora. Of particular significance is the capacity of numerous metabolites produced by the gut microbiota to modulate the activity and hormonal secretion of enteroendocrine cells. This review examines the effects of dipeptidyl peptidase 4 (DPP-4) inhibitors, glucagon-like peptide 1 (GLP-1) receptor agonists and GLP-1/gastric inhibitory polypeptide (GIP) receptor dual agonists on the composition of the gut microbiota in both mice and human subjects. The nature of this interaction is complex and bidirectional. The present study demonstrates the involvement of the incretinic axis in modulating the microbial composition, with the objective of providing novel preventative strategies and potential personalised therapeutic targets for obesity and T2DM.

## 1. Introduction

Obesity is regarded as a global epidemic, with prevalence figures exceeding 2 billion. The prevailing definition of obesity, as outlined by the World Health Organization, is a body mass index (BMI) of 30 kg/m^2^ or above [1]. However, it is important to note that obesity is not solely defined by the BMI; rather, it is a complex condition influenced by individual factors, including genetic and epigenetic influences, and lifestyle factors such as unhealthy diet, overeating, and low physical activity [2]. This complex condition is associated with an elevated risk of several chronic conditions, including diabetes, hypercholesterolemia, cardiovascular disease (from hypertension to heart failure), and cancer [3]. Type 2 diabetes mellitus (T2DM) is a prevalent chronic metabolic disorder, characterised by hyperglycaemia resulting from a combination of insulin resistance and inadequate insulin secretion [4,5,6,7]. The diagnosis of T2DM is typically made on the basis of fasting plasma glucose values, 2 h plasma glucose values derived from a 75 g oral glucose tolerance test, or haemoglobin A1C (HbA1c) levels [5]. It is well established that carrying excess weight, or being obese, is a major risk factor for the development of T2DM [8,9]. The prevalence and incidence of both obesity and T2DM are steadily increasing and reflect each other. An unhealthy diet, characterised by a high consumption of carbohydrates and lipids, and a low intake of fibre, is a major contributing factor to the development of obesity and, potentially, T2DM. In individuals with T2DM and a BMI in the overweight or obese range, weight reduction has been shown to enhance glycaemic control and decrease reliance on glucose-lowering medications [9]. The management of T2DM necessitates a multifaceted, person-centred approach. The promotion of healthy lifestyle behaviours and diabetes self-management is to be emphasised alongside any pharmacotherapy [10].

Furthermore, obesity, insulin resistance and type 2 diabetes mellitus have been largely correlated to a reduction in bacterial load and diversity, resulting in a condition known as intestinal dysbiosis. The recent emergence of novel antidiabetic medications has been demonstrated to exert a favourable influence on the composition of the intestinal microbiota. In this review, the extant data on the bidirectional interactions between the gut microbiota and incretin-based therapies, including GLP-1R agonists, DPP-4 inhibitors and GLP-1/GIP receptor dual agonists, are summarised.

## 2. Incretins and Incretin-Based Therapies

**Figure 1:**
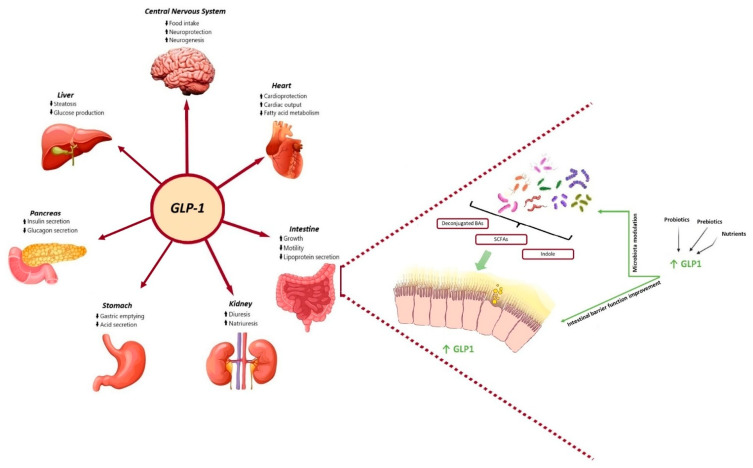
Physiological benefits of GLP-1 and gut microbiota metabolites and their bidirectional interaction. On the one hand, GLP-1 promotes insulin synthesis and secretion in pancreatic beta cells and then improves glucose homeostasis, delays gastric emptying and reduces gastric acid secretion, reduces intestinal motility and lipoprotein secretion, promotes diuresis and natriuresis, increases liver glycogen storage and decreases liver sugar output, and suppresses appetite in the hypothalamus of brain. On the other hand, SCFAs, secondary bile acids and indole, have been shown to directly stimulate the release of incretins from intestinal L-cells and represent the key candidates involved in the crosstalk between microbes and host cells. Microbial-based therapeutics, including probiotics, nutrients and prebiotics, could directly target the gut microbiota or act as an adjunctive to the GLP-1 receptor agonist to restore the balance of several certain dysbiotic gut microbiota and improve gut barrier function, therefore improving glycaemic control and glucose tolerance.

Incretin-based therapies represent a novel treatment for both T2DM and obesity, relying on the insulinotropic actions of the gut hormone glucagon-like peptide-1 (GLP-1) and, most recently, on the combined action of GLP-1 and the gastric inhibitory polypeptide (GIP) hormones. Incretins are gastrointestinal hormones that promote postprandial insulin secretion in a glucose-dependent manner. There are two types of incretins: GLP-1 and GIP. Both are secreted by intestinal cells in response to meals, with GLP-1 being secreted from L cells that are found in the large and lower bowel and GIP from K cells in the upper bowel [11]. There is a specific threshold, between 1 and 2 kcal/min, for glucose-induced GLP-1 secretion in the small intestine, while the secretion of GIP is more sensitive [12]. GLP-1 subsequently binds to GLP-1 receptors (GLP-1R), which are widely expressed in multiple organs and tissues, including the gastrointestinal tract, the endocrine pancreas, the heart, and the central nervous system, thereby exerting pleiotropic functions (Figure 1).

**Figure 2:**
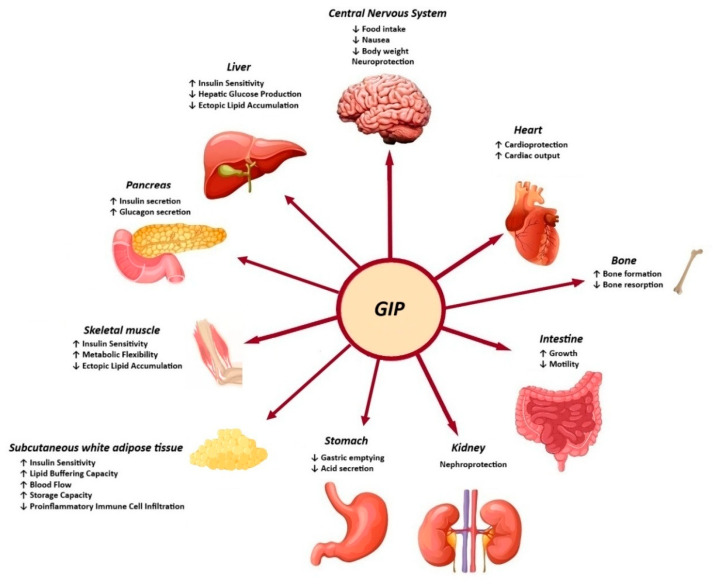
Effects of GIP in peripheral tissues.

The biological action of GIP is promoted through binding to the GIP receptor (GIPR), a class B G-protein-coupled receptor that is similar to GLP-1R and belongs to the glucagon receptor family. The GIPR is expressed in the endocrine pancreas, adipocytes, myeloid cells, the endothelium of the heart and blood vessels, the inner layers of the adrenal cortex and the central nervous system. The role of GIP in regulating energy balance is achieved through cell surface receptor signalling in the brain and adipose tissue [13]. It is evident that both GLP-1 and GIP play a substantial role in the function of beta cells, including the promotion of increased beta-cell mass, enhanced insulin secretion, and the preservation of beta-cell survival [14]. Moreover, the GIP-dependent reduction in insulin clearance contributes to the enhancement of peripheral insulin levels, thereby ensuring the maintenance of normal blood glucose levels (Figure 2) [15].

However, GLP-1 and GIP are rapidly degraded in plasma by dipeptidyl peptidase IV (DPP-4), resulting in a very short half-life of approximately 1.5 min [16,17,18].

**Figure 3:**
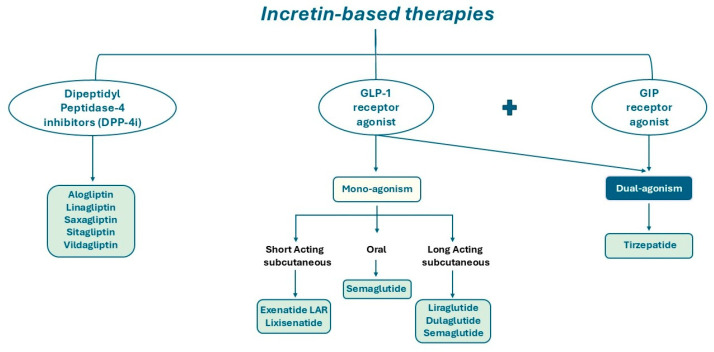
Schematic illustration of DPP-4 inhibitors, mono-agonists and dual-agonists of GLP-1 and GIP receptor.

DPP-4 is a type II integral transmembrane glycoprotein with enzymatic activity; it also exists in a soluble form in the plasma, lacking the cytoplasmic and transmembrane domains [19,20]. The primary substrates for DPP-4’s catalytic activity are incretins responsible for glucose metabolism, including GIP, GLP-1 and GLP-2 [21,22,23]. Consequently, DPP-4 inhibitors may have a pivotal role in prolonging the circulating half-life of GLP-1 and GIP, thereby preventing their cleavage [24]. Indeed, DPP-4 inhibitors represent a therapeutic approach for the management of patients with T2DM (Figure 3).

These inhibitors reduce blood glucose levels, enhance glycated haemoglobin levels, and do not induce hypoglycaemia. There is evidence that the rate of gastric emptying (GE) may influence the magnitude of effect on glucose-lowering to DPP-4 inhibition. Indeed, the ability of DPP-4 inhibitors to reduce postprandial glycaemia increases when GE is faster, partly because this leads to enhanced GLP-1 secretion, but also because higher postprandial glucose levels are necessary for GLP-1-mediated insulin secretion and glucagon suppression [25,26].

Moreover, nutritional strategies may improve the lowering of blood glucose levels induced by DPP-4 inhibitors. A promising approach to optimise DPP-4 inhibitor action involves the administration of a small quantity of a specific macronutrient at a fixed interval before a meal. The presence of nutrients in the small intestine induces the release of gut peptides, including endogenous GLP-1 and GIP; these peptides improve the glycaemic response to the subsequent meal and slow gastric emptying [27,28].

DPP-4 inhibitors have been shown to improve β-cell function and inhibit α-cell secretion, with the potential to enhance insulin sensitivity, as evidenced by increased β-cell mass in animal studies.

Furthermore, many studies demonstrated that DPP-4 gene-deficient mice exhibited enhanced postprandial glucose control and resistance to developing hyperinsulinemia and obesity. Exogenous DPP-4 inhibitors improve glucose tolerance in wild-type mice, but not in DPP-4 knockout mice [29]. Beyond its catalytic functions, DPP-4 may also regulate the immune system through the cleavage of various chemokines and cytokines, such as erythropoietin, stromal cell-derived factor-1, granulocyte colony-stimulating factor, Interleukin-3, and granulocyte-macrophage colony-stimulating factor [30]. Additionally, DPP-4 exert non-catalytic functions, including the reduction in dendritic cell/macrophage-mediated adipose tissue inflammation in obesity by affecting macrophage migration (CD11b+, CD11c+, and Ly6Chi) [31,32]. Moreover, DPP-4 enhances regulatory T cell (Treg) expansion and transforming growth factor beta levels in non-obese diabetic mice whilst concomitantly promoting T-cell activation via its interaction with adenosine deaminase [33,34].

GLP-1R agonists used in clinical settings (Figure 3) are structurally modified to exhibit relative resistance to DPP-4 cleavage, resulting in a long-circulating half-life [22,35].

The rapid cleavage of the N-terminal His–Ala residues of GLP-1 by DPP-4 expressed on surrounding tissues results in the inactivation of GLP-1 and a consequent short half-life [19]. GLP-1 analogues exert various pharmacological actions by increasing GLP-1 levels 10-fold or more. GLP-1R agonists have been shown to restore the sensitivity of β-cells to insulin, induce insulin secretion [19], and modulate body weight loss by potentially inhibiting GE [36]. In the central nervous system, GLP-1R agonists regulate glycaemic control and possess a protective effect on neuronal damage [37]. Furthermore, GLP-1 may also act on the heart and gut, directly or indirectly through the sympathetic nervous system, to regulate heart rate and gut peristalsis via the nitric oxide-mediated suppression of intestinal motility [38,39,40]. Common adverse effects include nausea, diarrhoea and constipation [41]. The relationship between intestinal norepinephrine and GLP-1 remains to be fully elucidated [11]. The acute stimulation of the β-adrenergic receptor by isoproterenol induces a significant increase in the secretion of GLP-1 and PYY. This suggests that the activation of β2-adrenergic receptors on intestinal L cells could promote the release of these hormones [42]. At the same time, it has been observed that a prolonged increase in catecholamine production may inhibit GLP-1 secretion [43].

Recently, a new pharmaceutical agent was developed, which has the capacity to simultaneously co-activate both the GIPR and the GLP-1R (Figure 3). Tirzepatide, a drug that has been hailed as a pioneering innovation in its field, was shown to improve glycaemic control by increasing insulin sensitivity and lipid metabolism and reducing body weight. The co-activation of these two receptors enhances β cell function, offering a more effective treatment for diabetes and obesity with a reduced adverse effect profile compared to selective GLP-1R agonists. Until now, clinical trials have demonstrated the remarkable effectiveness of this compound, once-weekly injected, in achieving optimal glycaemic control and substantial weight reduction [44,45]. The magnitude of the effects of tirzepatide on glycaemia and weight loss marks the commencement of a new era in diabetes therapy, with the potential to treat a significant proportion of patients according to currently established targets.

Even the SURMOUNT-3 study evaluated the efficacy and safety of tirzepatide in the treatment of obesity. This study demonstrated that the combination of tirzepatide with intensive behavioural therapy resulted in significant weight loss, with a mean reduction of 25.3% after 84 weeks of treatment, a result that is superior to the 20.9% weight loss reported in the SURMOUNT-1 study at week 72, which used tirzepatide alone without intensive behavioural therapy. The sequential therapy (intensive behavioural therapy followed by tirzepatide) may have an additive effect on weight loss, but further studies are needed to confirm this result. But the main study’s results highlight the importance of combining new obesity medications with lifestyle modification interventions to achieve optimal results [46].

## 3. Gut Microbiota

In the course of its development, the human gastrointestinal tract accumulates a dense, varied population of microorganisms called ‘microbiota’. The bacteria populating human intestines are in the process of evolving and coevolving with their host in a system of beneficial symbiosis [47].

**Figure 4:**
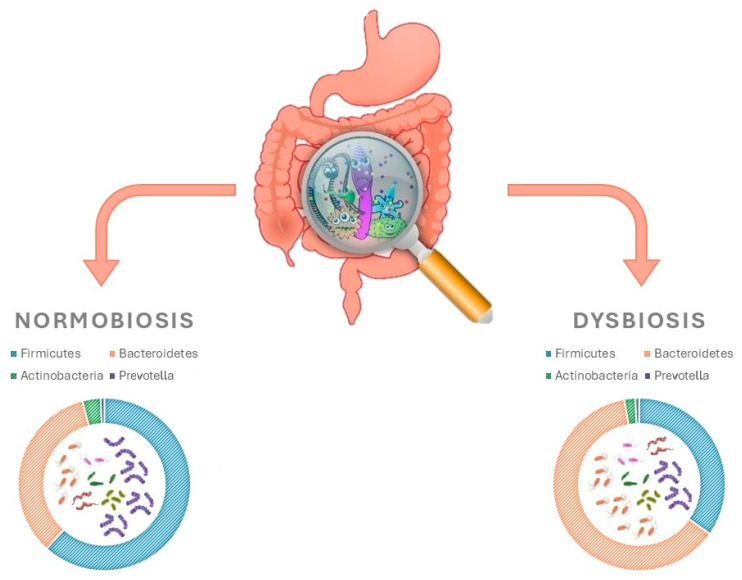
Gut microbiota and how they differ in intestinal homoeostasis vs. dysbiosis.

Until a decade ago, information available on the human microbiota was based upon traditional culture techniques. More recently, the use of genomic sequencing methods has enhanced the capabilities of studying the gut microbiota [47,48]. Numerous fragments from MetaHit and Human Microbiome Project databases have contributed towards building a better and more exhaustive understanding of the microorganisms present in the human gut [49,50,51]. In a typical adult, gut microbiota composition includes fungi, viruses, and parasites in addition to bacteria. The total number of species of microbes in the gut microbiota can reach up to 5000, with a combined weight of approximately 2000 g. This is equivalent to 10 times the amount of microbial cells found in the entire human body [52]. Studies that have been carried out have contributed to the identification and isolation of 2172 bacterial species, which have been subsequently categorised into 12 different phyla. The main species are, in order of abundance, Firmicutes (Ruminococcus, Clostridium and Eubacterium), Bacteroidetes, Actinobacteria and Prevotella (Figure 4) [52,53,54].

Features of the small intestine such as high levels of acids, oxygen, and antimicrobials and a short transit time inhibit the growth of bacteria [47,51]. Thus, in this environment, only facultative anaerobes can survive, which rapidly grow and are membranous epithelium-adhering and permeable to oxygen [47,51]. On the contrary, the shallow characteristics of the large intestine support the existence of a highly diverse microbial community, predominantly anaerobes trained on complex carbohydrates and polysaccharides not utilised in the small intestine [47,51].

The microbiota present within the gastrointestinal tract are understood to exert a pivotal influence on host physiology, engaging in interactions with both the endocrine and immune systems within the gastrointestinal tissues.

The bacteria within the gut microbiome are involved in many biological processes, including the harvesting of energy from food (degrading fibres and complex polysaccharides) and the manufacturing of neurotransmitters (e.g., serotonin, vitamins and enzymes). The production of microbial metabolites can be categorised into three distinct pathways: the first involves the fermentation of food elements by intestinal microorganisms, such as 2-oleoyl glycerol (derived from dietary fats); the second pathway entails the direct production of microbial metabolites by the intestinal microbiota, including lipopolysaccharides (LPSs); and the third pathway involves the synthesis of these microbial metabolites by the host, followed by subsequent modification by bacteria. In addition, the gut microbiota protects the body from pathogens with a competitive mechanism and through the production of antimicrobial substances. In summary, the intestinal microbiota modulate the immune system and help maintain the integrity of the intestinal barrier by strengthening tight junctions between enterocytes and stimulating mucus production [52].

Nutritional intake exerts a profound influence on the equilibrium between beneficial and opportunistic microbial composition, with dietary modifications capable of affecting this ratio [52]. In addition to nutrient availability, various factors influence fluctuations and stabilities of bacterial levels, such as osmolality, pH, temperature, hormones, neurotransmitters derived from the host, cytokines, and viruses that infect bacteria (bacteriophages) [55,56,57,58]. Advances in sequencing technologies and population-scale studies have revealed that the microbiome assists in the expansion of host genomes by facilitating the host’s physiology and metabolism and associating with several diseases [59,60]. An imbalance of the microbiota called dysbiosis has been associated with numerous diseases, including obesity, diabetes, cardiovascular diseases, chronic inflammatory bowel diseases, autoimmune diseases and neurological disorders [61].

## 4. Incretin-Based Drugs and Gut Microbiota

Incretin-based therapies represent a novel treatment for both T2DM and obesity, relying on the insulinotropic actions of the gut hormone GLP-1 and, most recently, on the combined action of GLP-1 and the GIP hormones. The gut microbiota is a complex community consisting of more than 500 microbial species and it is involved in many biological processes, including energy metabolism, inflammatory response, immunity and gut–brain neural circuits. Despite the numerous preclinical and clinical studies conducted on the interaction between incretin-based drugs and gut microbiota, there are still several aspects of this interaction that are not yet fully understood.

### 4.1. Preclinical Studies

A number of studies conducted on mice have indicated that both DPP-4 inhibitors and GLP-1-R agonists have the capacity to modify the bacterial composition of the intestinal microbiome. The primary outcome of interest pertained to the variation in the balance between Firmicutes and Bacteroidetes. In a study by Yan et al. [62], it was demonstrated that a high-fat diet led to a reduction in Bacteroidetes and an increase in Firmicutes and Tenericutes. The administration of sitagliptin resulted in the reversal of these gut microbiota changes and the modification of a set of bacteria producing short-chain fatty acids (SCFAs) in rats with T2DM who were fed a high-fat diet [62].

In a similar vein, other studies have shown that the use of vildagliptin led to an increase in Bacteroidetes and a decrease in Firmicutes, a decrease in the Firmicutes/Bacteroidetes ratio, and an increase in butyrate-producing bacteria in obese and diabetic mice (Figure 4) [63,64]. Moreover, it has been reported that vildagliptin can exert positive effects through the variation in the intestinal microbiota by increasing *Lactobacilli* spp., promoting the production of propionate and decreasing *Oscillibacter* spp. [65]. In order to elucidate the mechanisms by which vildagliptin modifies the intestinal microbiota, the experiments conducted demonstrated the drug’s ability to indirectly reduce the hepatic gene expression of proinflammatory cytokines, reduce Toll-like receptor ligands in caecum contents, promote the expression of antimicrobial peptides, and restore the depth of the ileum crypts [65]. Furthermore, Liao X. et al. and Silva-Veiga et al. showed that sitagliptin and linagliptin, respectively, increase the abundance of succinate and Bacteroidetes in non-diabetic mice [66,67].

In order to evaluate the hypothesis that alterations of the gut microbiota induced by DPP-4 inhibitors treatment improve glucose homeostasis, a comparison has been made of the effects of DPP-4 inhibitors with those of α-glucosidase inhibitors. While both altered the composition of the gut microbiome, the hypoglycaemic effect was attributed to the modulation of the gut microbiome by DPP-4 inhibitors. Specifically, these drugs reversed the changes in 68.6% of intestinal bacteria genera induced by a high-fat diet. Furthermore, a study on faecal microbiota transplantation demonstrated that DPP-4 inhibitors led to a favourable alteration in the microbiome and an enhancement in glucose tolerance in colonised mice, while acarbose did not exhibit this effect [66].

However, a separate preclinical study involving 60 C57BL/6 ApoE/mice (half of which received streptozotocin) and randomised to 8 weeks of treatment with liraglutide or saxagliptin revealed that only the former substantially modified the composition of the intestinal microbiota, particularly the concentration of phylotypes relevant to weight [68].

The acute administration of liraglutide (a GLP-1R agonist) to mice increased both caecal levels of the caseinolytic protease B, a component of Escherichia coli, and norepinephrine. However, these events were blocked by chemical sympathectomy. Furthermore, in vitro studies revealed that norepinephrine was found to permeate the intestinal lumen, and the c-Fructooligosaccharides (Fos) staining of the intermediolateral nucleus was interpreted as indirect evidence of intestinal tract sympathetic nervous system activation by GLP-1R agonists. Under normal conditions, the increase in Escherichia coli did not affect the host. However, in murine models of colitis, a bacterial translocation, accompanied by the attenuation of tight junction gene expression, was observed [69].

The administration of semaglutide, a long-acting GLP-1R agonist, has been demonstrated to modify the bacterial composition of the intestinal microbiome. A preclinical study demonstrated that a high-fat diet in mice induced a significant increase in Lachnospiraceae and Bacteroides and a significant decrease in Akkermansia. The use of semaglutide has been demonstrated to mitigate microbial dysbiosis, restore lost flora and suppress excessive bacterial abundance [70,71]. In a similar manner, in a mouse model of non-alcoholic fatty liver disease (NAFLD) with a db/db genetic background, semaglutide modified the gut microbiota, influencing Alloprevotella, Alistipes, Ligilactobacillus and Lactobacillus, and improved the integrity of the gut barrier [72]. Finally, in a mouse model of T2DM, semaglutide increased Bacterioidetes while significantly decreasing the abundance of Firmicutes [73,74].

In polycystic ovary syndrome (PCOS) mice, both semaglutide and liraglutide modulates both the alpha and beta diversity of the gut microbiota. Liraglutide increases the Bacillota-to-Bacteroidota ratio through up-regulating the abundance of butyrate-producing members of Bacillota, such as Lachnospiraceae. Conversely, semaglutide increases the abundance of Helicobacter [75].

Of particular interest was the observation that the gut microbiota composition of obese mice became phylogenetically similar after treatment with liraglutide or with a dual GLP-1/GLP-2 receptor agonist, thus suggesting that GLP-2 receptor stimulation played a minimal role in the modulation of the gut microbiome [76]. The observed changes were primarily characterised by variations in the distribution of the phelotypes of Proteobacteria and Verrucomicrobia, without, however, modifying the proportion of Firmicutes [76].

Moreover, liraglutide treatment was observed to moderate glucose intolerance and insulin sensitivity in a dose-dependent manner in diabetic male rats. Furthermore, it was demonstrated that both the diabetic state and liraglutide administration profoundly changed the composition of intestinal microbiota. The pyrosequencing of the V3-V4 region of the 16S rRNA genes revealed a significant alteration in the structure of the gut microbiota in male rats treated with liraglutide in comparison to male rats with diabetes. Specifically, the study noted a selective enhancement of SCFA-producing bacteria, including Bacteroides, Lachnospiraceae, and probiotic bacteria, such as Bifidobacterium, in male rats treated with liraglutide [77]. Finally, liraglutide was shown to enhance glucose-induced insulin secretion through the increase in the Bacteroidetes-to-Firmicutes ratio, which was achieved by enhancing certain immune cells (regulatory T cells and innate lymphoid cells 1 and 3) and by reducing the Th1 cell frequency [78]. In an experiment involving a mouse model of NAFLD, liraglutide treatment resulted in a change in the gut microbiota, reducing the Helicobacter genus and Proteobacteria phylum, and weight loss, consequently improving glucose homeostasis [79].

Yuan et al. hypothesised that GLP-1R agonists could help to modify the abundance and diversity of the gut microbiota, thus restoring balance in the gut microbiota [80]. Specifically, the study noted an enhancement in several SCFA-producing bacteria, including Bacteroidetes, Lachnospiraceae, and probiotic bacteria such as Bifidobacterium, in diabetic male rats treated with liraglutide compared to those given a placebo [80]. However, in another experiment, a significant weight reduction was associated with an increase in the ratio of Firmicutes to Bacteroides, regardless of glycaemic status, in both obese and diabetic subjects [81]. Furthermore, liraglutide treatment increased the abundance of intestinal Akkermansia muciniphila in mice [79,82,83].

As previously reported, tirzepatide is a dual agonist of GIP and GLP-1R, and it has been approved by the Food and Drug Administration for the treatment of T2DM [84]. Preliminary data indicate that tirzepatide exerts a regulatory influence on the gut microbiota and bile acid (BA) metabolism in diabetic mice [85]. Notably, tirzepatide improves the abundance of beneficial genera such as Akkermansia while concomitantly reducing farnesoid X receptor (FXR) expression in intestinal tissues and elevating the ratio of FXR antagonists (glycoursodeoxycholic acid, β-muricholic acid, hyodeoxycholic acid, and ursodeoxycholic acid) to natural agonists (cholic acid, lithocholic acid, chenodeoxycholic acid, glycocholic acid and taurodeoxycholic acid) [85].

Furthermore, tirzepatide treatment restores intestinal barrier integrity, mitigates possible endotoxemia through anti-inflammatory signalling pathways, and reverses intestinal dysbiosis in an obese, diabetic ovariectomised mice model [86]. A number of studies have demonstrated that GLP-1R agonism reduces systemic inflammation and alleviates experimental gut inflammation [87,88,89]. However, the actions of GIP in reducing inflammation in different tissue compartments are not as well understood. In preclinical studies, Hammoud R. et al. demonstrated that activation of GIPR signalling attenuates 5-fluorouracil-induced gut inflammation, whereas the loss of the GIPR exacerbates the extent of gut inflammation in the murine small intestine [90]. While these findings are preliminary, they suggest the potential of GIP to play a physiological and pharmacological role in response to gut injury.

### 4.2. Clinical Studies

Conversely, the existing body of human research is limited and frequently yields contradictory results. For instance, Ying X et al. demonstrated that liraglutide significantly increases the diversity of the gut microbiota, particularly Bacteroidetes, Bacilli and Proteobacteria [91]. However, a subsequent study, which included 37 T2DM patients who had been administered metformin, demonstrated that those who had been given liraglutide (after metformin had been ceased) exhibited significantly different microbiota compositions to those who had been given metformin [86]. The latter group also exhibited a greater number of genus Akkermansia bacteria [92]. The precise causative agent remains uncertain as metformin is recognised for its influence on the gut microbiota [93]. On the other hand, a novel randomised controlled trial suggested that the 12-week treatment with either the GLP-1R agonist liraglutide or the DPP-4 inhibitor sitagliptin, when used as add-on therapy to metformin or sulfonylureas, induced beneficial effects on glucose metabolism, body weight, and BA production, with no change in the alpha or beta diversity on faecal microbiota [94]. Additionally, a fixed combination of degludec and liraglutide administered over a period of six months in very old subjects with T2DM showed no change in gut microbiota structure [95]. A pilot study, performed on 52 subjects with T2DM, evaluated the association between GLP-1R agonist treatment (liraglutide and dulaglutide) and intestinal flora [96]. The subjects were divided into efficacious and non-efficacious groups based on whether GLP-1R agonist administration was efficacious or not after 12 weeks of treatment. The subsequent beta-diversity analysis revealed significant disparities in the gut flora between these two groups. The authors observed a negative correlation between the abundance of Prevotella, Bacteroidales, Ruminococcaceae, Dialister succinatiphilus, Eubacterium coprostanoligenes, Mitsuokella, Alistipes obesi, Mitsuokella, and Lactobacillus mucosae and insulin resistance. Conversely, a positive link was observed between the abundance of Butyricicoccus and Lachnoclostridium and a decrease in blood glucose levels. The study indicated a close association between GLP-1R agonist treatment and intestinal flora in T2DM [96]. Liang L. et al. have recently evaluated the structural changes in the composition of the intestinal flora of T2DM subjects after 1 and 48 weeks of dulaglutide therapy. The administration of dulaglutide for a single week did not result in any significant alterations in the intestinal flora. However, after 48 weeks of dulaglutide, a significant change in the composition of the intestinal flora was observed, with a substantial reduction in the abundance of intestinal flora [97]. Furthermore, the authors demonstrated a close association between fasting glucose levels, HbA1c levels, and the BMI on the one hand and changes in intestinal flora on the other [97].

## 5. Gut Metabolites and Incretins

A number of studies have shown that gut microbiota metabolites, including SCFAs and/or indole, can directly stimulate the release of incretins from colonic enteroendocrine cells (EECs) (Figure 1). This influences host satiety and food intake.

SCFAs play a pivotal role in the communication between host cells and microbes, directly stimulating the release of GLP-1 from enteroendocrine cells. The induction of cell-specific signalling cascades by SCFAs is achieved through the activation of specific receptors. Two distinct receptor types, designated as free fatty acid receptor (FFAR) 3 and FFAR2 (also termed G-protein-coupled receptor (GPR) 41 and GPR43, respectively), have been identified on EECs [98,99]. The activation of these receptors enhances GLP-1 secretion through increased intracellular calcium [100].

### 5.1. Preclinical Studies

Preclinical studies have demonstrated that GPR43 protects mice from obesity and hyperphagia. Moreover, GPR43-deficient mice exhibit impaired glucose tolerance on a normal diet, a consequence of the role that SCFAs and GPR43 play in the release of intestinal hormones [98]. Tolhurst et al. proved that in GPR43 knockout colonic tissue from mice, SCFAs-induced GLP-1 secretion was attenuated, while in mice knockout for GPR43, SCFAs did not lead to increased GLP-1 secretion [100].

It has been hypothesised that the fermentation of non-digestible carbohydrates in the gut could promote the differentiation of L-cells in the intestine, also increasing their numbers. A study conducted on rats found that administration of nondigestible carbohydrates results in an increase in the number of enteroendocrine L cells in the proximal colon, resulting in increased GLP-1 production (mediated by SCFAs) [101]. Psichas et al. showed clearly that the intra-colonic injection of short-chain fatty acid propionate led to a significant increase in circulating plasma levels of GLP-1 and anorexigenic gut hormone peptide YY (PYY) in anaesthetised rats compared to the saline control [102].

SCFAs are produced by intestinal bacterial fermentation from dietary prebiotic fibres, such as FOS and inulin. Several studies revealed that the oral administration of FOS and inulin in rats is associated with improved glucose tolerance and hepatic insulin sensitivity through increased GLP-1 secretion, mediated by increased SCFAs [103].

### 5.2. Clinical Studies

Human-based studies appear to substantiate the correlation between the gut microbiota fermentation of non-digestible carbohydrates and incretin secretion. Piche T. et al. reported that the administration of a low-residue diet (i.e., 10 g fibre/day) in nine patients within reflux disease was associated with a significant increase in plasma GLP-1 after a meal [104]. The relevance of fermentation arising from the large intestine to the release of GLP-1 and lowering of postprandial glycaemia were also well supported in a clinical trial on T2DM patients, where GLP-1 release was indirectly quantified by the resultant hydrogen production in breath samples [27]. Conversely, the ingestion of lactulose (20 g/day) or the intracolonic administration of SCFAs in healthy volunteers has been associated with an increased production of incretins [105].

Furthermore, Cani P.D. et al. conducted a study in which 10 healthy adults were enrolled and divided into two groups. Each group was administered either prebiotic fibre or maltose dextrin for a period of two weeks [106]. The results of this study indicated that prebiotic administration was associated with increased blood GLP-1 and PYY and improved plasma responses to postprandial hyperglycaemia [106]. Although several studies suggest that the gut microbiota may play an important role in GLP-1 secretion through the production of SCFAs, additional studies are required to determine whether alterations of the gut microbiota are responsible for these effects.

In addition to SCFAs, the secretory activity of EECs can be modulated by several gut bacteria metabolites, including secondary BAs and indole derivatives.

Secondary BAs regulate GLP-1 secretion through the action of Takeda G protein-coupled receptor 5 on intestinal L cells [107,108]. These acids inhibit GLP-1 secretion by a mechanism that involves the activation of the farnesoid X receptor (FXR) [109,110]. In a study conducted by Rune E. et al., the intraluminal administration of BAs in an anaesthetised rat resulted in the stimulation of GIP, GLP-1, insulin, and C-peptide secretion [111].

A similar outcome was observed in a human study that was single-blinded; ten patients with obesity and T2DM received an intrarectal infusion of taurocholate, and GLP-1, insulin and PYY were increased in a dose-dependent manner [112]. The same result was also obtained in healthy subjects; ten men received taurocholic acid enemas, which induced a dose-dependent increase in GLP-1 and PYY; the participants also reported an increase in satiety [113].

In another study, Tongzhi et al. divided 10 healthy subjects into two groups in a double-blind order [114]. In each group, a jejunal catheter was placed. In addition, a balloon was inflated to 30 cm beyond the pylorus with the aim of aspirating bile. The administration of taurocholic acid, a secondary bile acid, or a saline control, both with and without glucose, was conducted through the catheter. The results demonstrated that the administration of TCA in the small intestine led to an increase in GLP-1 and a significant reduction in small intestine glucose levels, as well as an increase in the peptide C/glucose ratio [114].

Another product of the gut microbiota is indole, which is produced from tryptophan. This molecule has been observed to exert a dual effect on GLP-1 release. Short exposures result in increased GLP-1 secretion in immortalised and primary mouse colonic L cells, whereas secretion is reduced following longer exposures. Consequently, indole could be considered a molecule through which the microbiota exert their influence on host metabolism [115].

## 6. The Relationship Between the Gut Microbiota and Incretin-Based Therapies: A Complex Bidirectional Interaction

The intestinal microbiota plays an important role in many biological processes, including gut barrier function, digestion, the modulation of the immune system and/or glucose metabolism. Obesity, insulin resistance, T2DM and metabolic syndrome have been largely correlated to a reduction in bacterial load and diversity, configuring a picture termed intestinal dysbiosis [116,117]. The ratio between the bacterial species Firmicutes and Bacteroidetes is widely regarded as playing a significant role in disease and health [60,61,118].

A multitude of metabolites produced by the gut microbiota modulate the activity of enteroendocrine cells and their hormonal secretion. Such metabolites include 5-hydroxytryptamine [119], indole [111], LPS [120], secondary BAs [99] and SCFAs [94]. These increase GLP-1 secretion, either directly or indirectly, through a variety of mechanisms [88,96,121,122,123,124]. Of particular interest is the role of SCFAs, which activate various receptors, including GPR41 (FFA3) and GPR43 (FFA2), expressed by enteroendocrine L cells. The activation of these receptors have been shown to enhance GLP-1 secretion through increased intracellular calcium [94,96,123]. Furthermore, the gut microbiota exerts a regulatory effect on GLP-1 secretion through Takeda G protein-coupled receptor 5 on intestinal L cells via secondary BAs metabolism [96,97]. Conversely, some metabolic diseases, including T2DM, are partly related to a decreased production of SCFAs [118,125], which are predominantly formed in the colon.

Moreover, a plethora of evidence suggests that the intestinal microbiota can not only influence incretin secretion, but in turn can also be influenced by incretin-based therapies and modulate the individual response to such drugs [96]. This interaction is complex and bidirectional. Numerous studies have indicated that the administration of DPP-4 inhibitors and the GLP-1R agonist could potentially modify the intestinal microbiota (Table 1).

The mechanisms by which DDP-IV inhibitors and GLP-1R agonists modify the composition of the gut microbiota are still not clear. As suggested by Zeng Y. et al. [126] and as reported in our manuscript (see Section 4.1 and Section 4.2, Preclinical Studies and Clinical Studies, respectively), DDP-IV inhibitors impact the composition and function of the gut microbiota and they correct the dysbiosis of the microbiota. It is hypothesised that DPP-4 inhibitors can normalise the faecal microbiota composition and promote a functional shift in the gut microbiome. Numerous studies have demonstrated that the administration of DPP-4 inhibitors exerts a substantial influence on the composition of the gut microbiota, as evidenced by an augmentation in the abundance of Bacteroidetes, a concomitant escalation in the production of succinate, and an amelioration of microbiota dysbiosis in obese and T2DM mice [66,67]. These changes may contribute to the observed beneficial effects. As previously reported, DPP-4 inhibitors improve glucose metabolism and increase plasma GLP-1 concentrations. However, the magnitude of GLP-1 secretion is dependent on the rate and load of nutrient entry to the small intestine, so the individual basal rate of gastric emptying may well explain the different response to DPP-4 inhibitors [27].

It is plausible that the shifts in gut microbiota could contribute to the modulation of glucose homeostasis by increasing GLP-1 levels.

Furthermore, liraglutide treatment increases the Firmicutes to Bacteroides ratio in diabetic mice with normal weight, thereby indicating that liraglutide has the capacity to modulate the gut microbiota composition in a positive manner. Collectively, the findings from animal studies imply that GLP-1R agonists and DPP-4 inhibitors may have a significant impact on the composition of the gut microbiota. Treatment with GLP-1R agonists increases the richness and diversity of the gut microbiota and changes the overall structure of the gut microbiota, especially some bacteria related to intestinal inflammation and glucolipid inflammation [76,77,91,127]. In addition, preclinical models suggest that changes in the gut microbiota induced by DPP-4 inhibitors and GLP-1R agonists could be linked to metabolic improvements, such as the attenuation of endotoxaemia and the increased production of short-chain fatty acids.

However, the paucity of human studies has yielded equivocal results to date. Additional studies and research are necessary due to the heterogenicity in human studies. Some human studies have suggested that incretin-based therapies do not result in any change to the structure of the gut microbiota using incretin-based therapies [94,95]. One potential explanation for this observation is that these medications are often administered in conjunction with other therapies, which may obscure the impact on the gut microbiota. Indeed, both DPP-4 inhibitors and GLP-1R agonists are invariably used in conjunction with metformin, and it has been observed that metformin dosage affects gut microbiota diversity [128]. It can be hypothesised that patients undergoing metformin treatment already possess a more favourable microbiota profile, thereby resulting in a diminished impact of DPP-4 inhibitors and GLP-1-R agonist therapies. Furthermore, these studies demonstrated that diet is the most significant factor in modulating the composition of the intestinal microbiota. The composition of the intestinal microbiota is influenced by the type of food consumed, which in turn affects the absorption of nutrients.

Especially, the Mediterranean diet increases Bifidobacterium, Faecalibacterium prausnitzii, Roseburia, and Lachnospiraceae (polyphenols and unsaturated fats appear to be the main drivers of the observed benefits); vegetarian diets increase microbial diversity and promote SCFA-producing bacteria like Akkermansia and Eubacterium; Ketogenic diets, in mouse models, improve insulin sensitivity and reduce inflammation, but their effects in humans are less clear; and Western diets reduce microbial diversity, increase gut permeability, and promote proinflammatory metabolites like TMAO (Trimethylamin N-Oxid) and LPS [129].

The microbiome plays a pivotal role in nutrient extraction, energy production, and low-grade inflammation, all of which have the potential to contribute to the development of obesity and T2DM [130,131].

As established, GLP-1R agonists induce a decrease in caloric intake. Consequently, there is a possibility that alterations to the gut microbiome may be observed if the diet is not standardised. Currently, few studies have directly compared different dietary profiles during incretin therapies. Most research focuses on the effect of incretin on the gut microbiota, without considering the interaction with specific dietary regimens. This lack of data makes it difficult to interpret discrepancies between clinical and preclinical trials, where dietary conditions can vary significantly.

In the context of animal experiments, the provision of a uniform diet to all subjects during the intervention period serves to mitigate the potential for confounding variables. This may provide a rationale for the observed discrepancies between animal studies and human trials. To fully understand the interaction between diet, gut microbiota and incretin-based therapies, studies are needed to compare directly different dietary profiles (e.g., the Mediterranean diet, ketogenic diet, vegetarian diet, Western diet, etc.) during treatment with incretins, evaluate changes in microbiota composition and functionality in response to specific combinations of diet and therapy, and analyse the metabolic and clinical effects resulting from these interactions.

Conversely, the increasingly frequent use of non-caloric artificial sweeteners (NASs) in diabetic and obese populations has led to their independent role in modifying the gut microbiota. Indeed, most of the NASs cross the gastrointestinal tract without being absorbed and therefore come into direct contact with the microbiota.

Suez et al. demonstrated that the consumption of NASs (firstly saccharin), both in mice and in humans, increases the risk of glucose intolerance, and changes in the composition of the microbiota in bacterial species are associated with the onset of type 2 diabetes in humans, first of all manifesting as the overexpression of Bacteroides [132].

However, recent studies have demonstrated a close association between GLP-1R agonist treatment and intestinal flora in T2DM subjects [98].

The changes in intestinal flora appear to be related to the modifications in metabolic parameters (e.g., fasting glucose levels, glycated haemoglobin levels, and BMI) induced by GLP-1R agonist therapies. Furthermore, Liang L. et al. reported that alterations in intestinal flora were time-related. Indeed, no significant changes in intestinal flora were observed after one week of GLP-1R agonist therapy in T2DM subjects; however, during long-term use, there was a significant change in the structure and overall composition of the flora and a significant decrease in the abundance of intestinal microorganisms compared to those before drug administration [97]. It is important to note that there is another relevant point of interaction between GLP-1 receptor agonists or tirzepatide and microbiota. This interaction is particularly significant in terms of their profound effects on both gastric emptying [133,134] and small intestinal transit [135]. Indeed, the inverse relationships in duodenal microbiota between the relative abundances of Streptococcus and Prevotella and the relative abundance of *Veillonella* spp with gastric emptying time is demonstrated. Consequently, this variation in gastric emptying and the exposure of nutrients to the small intestine warrants further investigations to delineate the underlying mechanisms and clarify its relevance to the risk of alterations in the gut microbiota [136].

Finally, several results show that alterations in the gut microbiota associated with metabolic diseases are different in men and women, and these characteristics may influence sex differences in the development and prevalence of metabolic diseases. Sex steroids, mainly oestrogen and testosterone, play a prominent role in the sexual dimorphism of gut microbiota [137]. Furthermore, there is recent evidence that the secretion of the GLP-1 may also be influenced by sex difference, probably through oestrogen signalling [138]. Therefore, in the complex interaction between the gut microbiota and incretin drugs, the interaction with sex hormones plays an important role, so the therapies may have sex-specific effects.

Despite numerous preclinical and clinical studies, the interaction between incretin drugs and the gut microbiota still shows several dark sides. Human data remain largely correlative, and correlations between improvements in metabolic parameters (e.g., reduced blood glucose levels, enhanced glycated haemoglobin levels, weight loss, improved hepatic steatosis) and specific microbial taxa have not established a causal link. Therefore, claims that DPP-4 inhibitors and GLP-1R agonists can significantly modulate the microbiota must be interpreted with caution, recognising that the observed changes may be secondary effects of metabolic improvement rather than primary therapeutic mechanisms.

To better understand the relationship between microbiota and drugs, multiomics techniques, such as metagenomics, metatranscriptomics, and metabolomics, should be applicated. These techniques enable the analysis of the composition and activity of the microbiota in a detailed manner, identifying specific patterns of gene expression and metabolic activity associated with drug response [139]. However, studies that have highlighted, through multiomics and strain-specific metabolite techniques, bidirectional interactions between the gut microbiota and incretin treatments are currently limited.

## 7. Conclusions

In conclusion, as demonstrated by several studies, incretin-based therapies have the capacity to influence the composition of the gut microbiota, thereby resulting in alterations to the bacterial flora. In addition, a lot of evidence suggests that the intestinal microbiota can not only influence incretin secretion, but in turn can also be influenced by incretin-based therapies and modulate the individual response to such drugs. The potential mechanisms by which the microbiota modulates drug metabolism and efficacy may involve the microbial-mediated metabolism of xenobiotics (oral drugs) and microbial metabolites (short-chain fatty acids and BAs). However, despite numerous preclinical and clinical studies, the interaction between incretin drugs and the gut microbiota still shows several dark sides. Taxonomic data are important, but gut microbiota–incretin crosstalk should be studied not only through metagenomic description (what the bacteria are) but also in the metabolomic direction (how they interact). An important limitation is the heterogeneity of clinical trials due to interindividual complexity (age, gender, and ethnicity), which includes dietary variability and polypharmacy.

Further research is necessary to determine whether targeting the gut microbiota could enhance the endogenous production of incretins.

## Figures and Tables

**Table 1:** Effects of DPP-4 inhibitors and GLP-1R agonist on gut microbiota composition.

Incretinis Drugs	Subjects	Changes in Gut Microbiota	References
DPP4 inhibitors	mice	Increased abundance of phyla Firmicutes and Bacteroidetes	Liao X et al., 2019 [66]
DPP4 inhibitors	diabetic rats	Increased Firmicutes and Tenericutes and as decreased Bacteroidetes	Zhang Q. et al., 2017 [63]
DPP4 inhibitors	type 2 diabetic high-fat diet-fed rats	High-fat diet increased Firmicutes and Tenericutes and decreased Bacteroidetes, and sitagliptin induced a reversal of the gut microbiota changes and modified a set of bacteria producing SCFA	Yan X. et al., 2016 [62]
DPP4 inhibitors	Western diet-fed mice	Increased *Lactobacilli* spp. and propionate production along with decreased *Oscillibacter* spp.	Olivares M et al., 2018 [65]
DPP4 inhibitors	obese mice	Increased the abundance of Bacteroidetes and succinate	Silva-Veiga FM ET AL., 2022. [67]
DPP4 inhibitors	metabolically dysfunctional mice	Decreased Firmicutes/Bacteroidete ratios and increased butyrate-producing bacteria	Ryan PM et al., 2020 [64]
GLP-1 receptor agonists and DPP4 inhibitors	mice	Liraglutide, but not with saxagliptin, increased ratio of Firmicutes to Bacteroides	Wang L et al., 2016 [68]
GLP-1 receptor agonists	simple obese and diabetic obese rats	Increased ratio of Firmicutes to Bacteroides	Zhao L et al., 2018 [81]
GLP-1 receptor agonists	diabetic male rats	Elevated SCFA-producing bacteria (Bacteroides and Lachnospiraceae) and Bifidobacterium	Zhang Q et al., 2018 [77]
GLP-1 receptor agonists	dysmetabolic mice	Increased frequency of Bacteroidetes to Firmicutes phyla Ratio	Charpentier J et al., 2021 [78]
GLP-1 receptor agonists	db/db mice	Increased abundance of intestinal Akkermansia muciniphila	Liu Q et al., 2020 [82]
GLP-1 receptor agonists	obese mice	Increased abundance of intestinal Akkermansia muciniphila	Moreira GV et al., 2018. [79]
GLP-1 receptor agonists	wild type mice	Increased abundance of intestinal Akkermansia muciniphila	Wang H et al., 2021 [83]
GLP-1 receptor agonists	diabetic rats	Increased Bacteroides–Firmicutes ratio	Yuan X et al., 2018 [80]
GLP-1 receptor agonists	obese mice	Varied distribution of phelotypes of Proteobacteria and Verrucomicrobia without modifying proportion of Firmicutes	Madsen MSA et al., 2019 [76]
GLP-1 receptor agonists	obese mice	Mitigated microbial dysbiosis induced by high-fat diet by impacting diversity of gut microbiota	Duan X et al., 2024 [70]
GLP-1 receptor agonists	db/db mice	Altered gut microbiota, especially Alloprevotella, Alistpes, Ligilactobacillus and Lactobacillus	Mao T et al., 2024 [72]
GLP-1 receptor agonists	obese mice	Abundance of gut microbiota changed, decreased Akkermansia, Muribaculaceae, Coriobacteriaceae, Clostridia and increased Romboutsia, Dubosiella, Enterorhabdus	Feng J et al., 2024 [71]
GLP-1 receptor agonists	diabetic rats	Changed gut microbiota profile, increased Bacterioidetes, Bacteroides acidifaciens, and Blautia coccoides	De Paiva IHR et al., 2024 [73]
GLP-1 receptor agonists	diabetic rats	Decreased abundance of Firmicutes, Actinobacteriota, and Lactobacillus and increased Bacteroides and norank_f_Muribaculaceae content.	Luo Y et al., 2024 [74]
GLP-1 receptor Agonists	PCOS mice	Modulated alpha and beta diversity of the gut microbiota	Xiong C et al., 2024 [75]
GLP-1 receptor agonists	humans	Increased genus Akkermansia bacteria	Wang, Z et al., 2018 [93]
GLP-1 receptor agonists	humans	Increased diversity and richness of gut microbiota, especially Bacteroidetes, Proteobacteria, and Bacilli	Ying X et al., 2023 [91]
GLP-1 receptor agonists and DPP4 inhibitors	humans	No change in alpha or beta diversity of gut microbiota when they were used as add-on therapies with metformin or sulfonylureas	Smits MM et al., 2021 [94]
GLP-1 receptor agonists	humans	No change in microbiome biodiversity or community	Rizza S et al., 2023 [95]
GLP-1 receptor agonists	humans	Change in microbiome composition, with significant reduction in abundance of intestinal flora	Liang L. et al., 2024 [97]

## Data Availability

Not applicable.

## Abbreviations

BAs	Bile acids
DPP-4	Dipeptidyl peptidase IV
EECs	Colonic enteroendocrine cells
FFAR	Free fatty acid receptor
FOS	Fructooligosaccharides
FXR	Farnesoid X receptor
GIP	Gastric inhibitory polypeptide
GIPR	GIP receptor
GLP-1	Glucagon-like peptide-1
GLP-1R	GLP-1 receptors
GPR	G-protein-coupled receptor
LPS	Lipopolysaccharide
NAFLD	Non-alcoholic fatty liver disease
PCOS	Polycystic ovary syndrome
PYY	Peptide YY
SCFAs	Short-chain fatty acids
T2DM	Type 2 diabetes mellitus
TMAO	Trimethylamine N-oxide
Treg	Regulatory T cell
NAS	Non-caloric artificial sweetener
